# Lessons learned? Increasing injury severity of electric-scooter accidents over a period of one year: a monocentric follow-up study at a level 1 trauma center

**DOI:** 10.1007/s00590-023-03583-1

**Published:** 2023-06-03

**Authors:** Jannik Leyendecker, Michael Hackl, Tim Leschinger, Jan Bredow, Felix Krane, Peer Eysel, Lars P. Müller, Andreas Harbrecht

**Affiliations:** 1 Department of Orthopedics and Trauma Surgery, Faculty of Medicine and University Hospital Cologne, University of Cologne, Cologne, Germany; 2 Department of Neurological Surgery, University of Washington, Seattle, WA USA; 3 Department of Orthopedics and Trauma Surgery, Krankenhaus Porz Am Rhein, University of Cologne, Cologne, Germany

**Keywords:** E-scooter-related accidents, Injury pattern, Prevalence, Monocentric

## Abstract

**Purpose:**

After major COVID-19 lockdown measures were suspended in 2021, E-scooter mobility regrew rapidly. In the meantime, multiple studies were published on the potential risks for e-scooter drivers and the necessity for wearing protective equipment. But did the drivers learn their lessons?

**Methods:**

We observed data of E-scooter-related accidents admitted to the emergency department of a level 1 German trauma center in the year 2021 and compared the data with our previous report (July 2019-July 2020).

**Results:**

*N* = 97 E-scooter-related accidents were included, marking a 50% increase when compared to the previous observation. Most patients were young adults (28.18 ± 1.13 years) with a notable shift towards a male population (25 vs. 63, *p* = 0.007). While the injury pattern remained unchanged, injury severity, reflected by a significant increase in shock room treatments (*p* = 0.005), hospital admissions (*p* = 0.45), and ICU admissions (*p* = 0.028), increased. Lastly, we report a higher injury severity of patients driving under the influence of alcohol, expressed by significant differences in hospital admissions, shock room treatments, ICU admissions, intracerebral bleeding (*p* < 0.0001), and injuries requiring surgery (*p* = 0.0017).

**Conclusion:**

The increase in injury severity and especially the substantial number of accidents due to driving under the influence of alcohol, are alarming for both trauma- and neurosurgeons. As the controversy surrounding the general use of E-scooters will continue, we urge representatives to intensify their efforts regarding prevention campaigns focusing on the potential dangers of E-scooters, especially when driving under the influence of alcohol.

## Introduction

Since their introduction in Germany on June 15th 2019 following their road approval, E-scooters have been subject of a controversial public debate [1]. Praised as the missing link in individual micro-mobility, allowing more people to commute via public transportation, hence reducing the number of cars in urban environments, the prevalence of E-scooters grew rapidly. Four years later, they shape the cityscapes of virtually every major city. Meanwhile, the legalization prompted safety concerns from medical professionals and law enforcement, stemming from a lack of mandatory helmets, an anticipated increase in accidents involving pedestrians and driving under the influence of alcohol [2–4].

Initial studies report a vast majority of minor injuries following E-scooter-related accidents. However, despite the substantial mobility restrictions due to the COVID-19 pandemic, E-scooter-related accidents have been surging up to sixfold within a few years [5–7]. Conflicting with a high occurrence of injuries to the head and face area, patients rarely use proper personal protective equipment [8–10].

As opposed to the intended incentivization for commuters to use public transportation, E-scooter-related accidents spike during nighttime and on the weekends, suggesting a higher incidence of accidents when used for recreational purposes. Additionally, they have been associated with a relevant prevalence of driving under the influence of alcohol or other substances that has been associated with a higher probability of traumatic brain injury, hospital admission and injury severity [3, 11–13].

Our previous reports align with the literature, indicating a growing number of E-scooter-related injuries after their introduction in 2019 [8]. Aim of this study was a critical reevaluation of this work with regards to injury prevalence, severity, the injury pattern, and the relevance of driving under the influence of alcohol after major lockdown measures due to the COVID-19 pandemic were suspended. Lessons learned? We expected a marked increase in E-scooter-related accidents with a comparable injury pattern.

## Methods

The conducted study was designed to be an analysis of retrospectively collected data. Additionally, we compared the collected data with our previously published report [8]. Included were patients > 14 years directly suffering from an E-scooter-related injury. Patients were included when admitted to the emergency department of our level 1 trauma center from 01/2021–12/2021. Patients suffering from accidents with an indirect involvement of E-scooters, i.e., stumbling over or being hit, as well as secondary in-patient visits at our clinic after an E-scooter related accident were excluded from the analysis. The involvement of an E-scooter-related injury was confirmed by the patients, their legal representative, as well as the presenting medical first-responders or law enforcement in case of reduced vigilance.

The treatment was at the discretion of the trauma surgeons on duty. Patients suffering from severe injuries were treated by an interdisciplinary team according to the national guidelines for polytrauma treatment [14].

Data was obtained from the patient charts and collected pseudonymously according to national law, and in accordance with the 1975 Declaration of Helsinki. Demographic parameters included sex, age, date of accident and worn protection gear. Laboratory findings were collected as well as performed imaging (X-ray, CT, MRI), injury pattern and treatment (surgery, inpatient). Primary endpoint was the assessment of all diagnosed injuries. Secondary endpoint was the rate of surgical treatment. A potential alcohol intoxication was confirmed via blood testing upon justifiable suspicion. The decision whether patients required treatment at an intensive care unit was at the discretion of the interdisciplinary shock-room team.

## Statistical analysis

Parameters are depicted as means ± standard error of the mean (SEM) for continuous or as number of cases with percentages for categorical variables. Continuous variables were analyzed using Student’s *t* test or Mann–Whitney *U* test for nonnormally distributed variables. Categorical parameters were compared using Chi-Square or Fisher’s exact test when appropriate. For the statistical analysis, a *p*-value < 0.05 was determined as statistically significant. We compared the collected data with the results from our previously published study [8].

Statistical calculations were carried out using Graphpad Prism (Version 9.5.0; GraphPad Software, Boston, MA). The guidelines of Strengthening the Reporting of Observational Studies in Epidemiology were implemented into this study’s design [15].

## Results

A total of *n* = 97 patients suffering from an E-scooter-related accident met the criteria to be included in this study. Mean age was 28.18 ± 1.13 with *n* = 63 (64.9%) of the patients being male and *n* = 34 (35.1%) being female (Table 1). The injuries were at the respective individuals’ fault in 89.7% of the cases. In only 2 (2.1%) cases, patients were wearing personal protection equipment (helmet, protectors). A total of *n* = 20 (20.6%) patients were tested positive for blood alcohol with a maximum of 2.5 per thousand and a minimum of 1.1 per thousand.

**Table 1:** Patient demographics, relevant imaging, hospitalization, and surgery data. If not declared differently, the values are shown in total cases (%). 2019-2020 data was obtained from our previously published report [8]. SEM = Standard error of the mean, CT = Computer tomography, ICU = Intensive care unit

Parameter (%)	2019–2020 (*n* = 59)	2021 (n = 97)	*p*-value
Sex			.007*
*Female*	34 (57.6)	34 (35.1)	
*Male*	25 (42.4)	63 (64.9)	
Age (SEM)	28.98 (1.2)	28.18 (1.13)	.534
Blood Alcohol	9 (15.3)	20 (20.6)	.403
Helmet worn	0 (0)	2 (2.1)	.527
Imaging performed	47 (76.6)*	85 (87.6)	.252
Whole body CT	3 (5.1)	25 (25.8)	.001*
Fractures	22 (37.3)	38 (39.2)	.866
*Multisite Fractures*	1 (4.8)	3 (7.9)	
Hospital admission	15 (25.4)	40 (41.2)	.045*
Shock room	3 (5.1)	26 (26.8)	.0005*
ICU admission	1 (1.7)	11 (11.3)	.028*
Surgery	10 (17.1)	17 (17.53)	.999

Statistically significant difference when compared to 2019/2020 (*p* = 0.05, Chi-square test)

Imaging was performed in 87.6% (*n* = 85) of the cases with X-rays being the most frequently used imaging method, followed by CT scans (*n* = 46; 47.42%). N = 25 of CT-scans were whole body trauma scans.

26.8% (*n* = 26) patients required an interdisciplinary shock-room treatment upon presentation. 41.2% (*n* = 40) patients were admitted to our hospital with 11.3% (*n* = 11) patients requiring ICU treatment. Main reason for ICU observation was intracerebral bleeding (ICB) in *n* = 7 (24.14%) cases. Fractures were reported in *n* = 38 (39.2%). Surgery was necessary in *n* = 17 (17.53%) (Table 1). No persisting neurological deficit was reported; however, one patient required an emergency splenectomy and another patient required cranial trepanation.

A total of 168 injuries were reported with the main injury location being the head accounting for a total of *n* = 86 (51.19%) injuries. Table 2 summarizes the injury patterns according to their respective anatomical region. With a total of *n* = 8, injuries to the face were operated most frequently, followed by a total of *n* = 5 injuries of the upper extremity.

**Table 2:** Injury Patterns and frequency of surgical intervention in E-Scooter related accidents (*n* = 59 for 2019 and *n* = 97 for 2021, respectively). 2019–2020 data was obtained from our previously published report [8]

Body part	Injury	2019–2020	Surgery	2021	Surgery
Head	**Total**	25 (42.37)	0 (0)	28 (28.86)	1 (1.03)
	Intracranial bleeding	1		7	1
	Skull fracture	0		2	
	Concussion	4		10	
	Contusion	10		5	
	Lacerations	10		4	
Face	**Total**	21 (35.59)	4 (6.78)	58 (59.79)	8 (8.25)
	Midface contusion	0		6	
	Midface fracture	1	1	11	4
	Nasal bone fracture	2	1	2	1
	Lower jaw fracture	3	2	5	3
	Anterior tooth trauma	10		14	
	Lacerations	5		20	
Chest	**Total**	0 (0)		10 (10.3)	0 (0)
	Pneumothorax			3	
	Rib fracture			4	
	Chest contusion			3	
Abdomen	**Total**	0 (0)		3 (3.09)	1 (1.03)
	Splenic laceration			2	1
	Renal laceration			1	
Spine	**Total**	4 (6.77)	0 (0)	7 (7.21)	0 (0)
	Cervical spine distortion	2		3	
	Thoracic spine contusion	1		1	
	Thoracic spine fracture	0		2	
	Lumbar spine contusion	1		1	
Upper extremity	**Total**	30 (50.84)	2 (3.39)	42 (43.30)	5 (5.15)
	Clavicular fracture	0		3	2
	AC-joint dislocation	0		1	
	Shoulder contusion	1		4	
	Shoulder dislocation	0		1	
	Proximal humerus fracture	0		1	
	Elbow fracture	8	1	6	1
	Elbow ligament damage	1		0	
	Elbow contusion	3		5	
	Forearm fracture	0		1	1
	Distal radial fracture	2	-	0	
	SL-ligament tear	0		1	1
	Wrist/hand contusion	10		16	
	Metacarpal fracture	1	1	3	
	Abrasions	4		0	
Pelvis	**Total**	1 (1.7)	0	3 (3.09)	1 (1.03)
	Pelvic contusion	1		2	
	Pelvic fracture	0		1	1
Lower extremity	**Total**	28 (47.45)	3 (5.08)	17 (17.52)	2 (2.06)
	Hip contusion	1		0	
	Knee contusion	10		6	
	Patella fracture	1		1	
	Proximal tibia fracture	1	1	0	
	Ankle fracture	1	1	4	2
	Ankle distortion	5	1	4	
	Metatarsal/toe fracture	2		1	
	Foot contusion	3	-	1	
	Abrasions	4		0	

Comparing the data to our previously published results revealed a change towards a male dominated patient population (*p* = 0.007). Unfortunately, changes regarding personal protection equipment were not reported (*p* = 0.527). However, an increasing injury severity was notable, indicated by a significantly higher number of inpatient treatments (*p* = 0.045), shock-room treatments (*p* = 0.0005) and ICU admissions (*p* = 0.028). Meanwhile, no change in the number of patients requiring surgery was observable (*p* = 0.999). Fortunately, no E-scooter related injury was fatal.

Focusing on patients driving under the influence of alcohol from both observed timespans revealed a higher injury severity for intoxicated drivers (Table 3). The latter showed a higher probability of hospitalization (*p* < 0.0001), and injuries necessitating surgery (*p* = 0.0017). Additionally, these patients required shock-room (*p* < 0.0001) and ICU treatment (*p* < 0.0001) more frequently. Lastly, these patients showed a significantly higher risk of ICB (*p* < 0.0001).

**Table 3:** Comparison of injury pattern and hospitalization data of patients driving under the influence of alcohol with non-intoxicated patients. 2019–2020 data was obtained from our previously published report [8]. ICU = Intensive care unit, ICB = Intracerebral bleeding

Parameter (%)	No Blood alc. (*n* = 127)	Intoxicated (*n* = 29)	*p*-value
Fractures	44 (34.65)	16 (55.17)	.0561
Hospital admission	30 (23.62)	25 (86.21)	< .0001*
Shock room	9 (7.09)	20 (68.97)	< .0001*
ICU admission	2 (1.58)	10 (34.48)	< .0001*
ICB	1 (0.79)	7 (24.14)	< .0001*
Surgery	15 (11.81)	11 (37.93)	.0017*

Statistically significant difference when compared to no blood alcohol (*p* = 0.05, Chi-square test)

## Discussion

Aim of our study was a critical reevaluation of our previously published data on E-scooter-related accidents. In our initial study, we attributed a relevant amount of the accidents to the insecurity of previously naive E-scooter drivers. As several studies elucidating the associated dangers of E-scooter utilization, especially when driving sans protective equipment, have been published in the meantime, the question on our mind was: Have the drivers learned their lessons?

E-scooters have established themselves as a new individual transport modality globally. As of today, they are omnipresent in urban environments. Their use is discussed controversially in both society and politics. Many people dislike a perceived clogging of the sidewalks due to thoughtless parking of the numerous scooters, while others praise them as an indispensable part of contemporary urban mobility in areas inaccessible via public transportation. Following a referendum receiving international media coverage, Paris elected a ban of shared E-scooters in the center of the city since its citizens felt the “littering” had gone too far, showcasing the current controversial debate regarding the use of E-scooters. Health care professionals represent an important discussion partner in this process as they provide expertise and education regarding the associated injury risks of E-scooter usage.

While the mortality of E-scooter-related accidents is comparably low, accounting for a total of 7 deaths in Germany for 01/2022–09/2022, as opposed to a total of 357 fatal bicycle injuries, several studies highlight the potential danger of E-scooter related accidents [12, 16–18]. Especially since, in spite of strict regulations, a sizeable number of injuries are due to an intoxicated driver [19]. Additionally, patients suffering from an E-scooter-related injury are more likely to require treatment at a major trauma center when compared to bicycles [20]. Due to their low center of gravity, and relevant speed (20 km/h), they are prone for accidents in short breaking situations with the driver wanting to prevent an imminent injury. Despite injuries frequently affecting the head and neck area, appropriate safety equipment (i.e., helmet) is only worn by a fraction [21–23].

In line with the literature, we report a substantial increase of E-scooter related accidents over the compared timespans, challenging our initial hypothesis of a relevant number of injuries occurring due to naivety of the drivers [24]. While no changes regarding patient age, personal protection equipment, fractures and concomitant surgeries were observable, the results indicate a higher injury severity of patients being admitted to the emergency department, reflected by a significant increase in hospital admissions, shock-room treatments, and ICU admissions (see Table 1). Additionally, driving under the influence of alcohol appears to be a major contributor to an increased injury severity. Patients driving under the influence of alcohol show markedly increased probabilities of requiring post-traumatic shock-room, inpatient or ICU treatment. Furthermore, they are more likely to suffer from fractures necessitating surgery. Lastly, the increased injury severity from patients driving under the influence of alcohol is showcased by the higher risk of suffering from ICB (Table 3).

The mechanical characteristics of E-scooters facilitate injuries of both the head and neck area [25]. These findings align with the observed injury patterns of our patients, indicating most injuries suffered being to either the face or the head. Uncharacteristically, we report a total of three abdominal traumas, one requiring an emergency splenectomy. Furthermore, three patients suffered from pneumothoraxes, with two necessitating an immediate decompression. Underlining our claim of a higher injury severity in intoxicated drivers, all patients suffering from abdominal trauma were intoxicated. Lastly, the relevant number of patients suffering from ICB in our study group showcase the potential dangers of E-scooter related accidents.

Four years after their introduction, E-scooters and the inevitable accidents will continue to challenge emergency departments globally. With regards to the injury pattern and the alarming number of injuries involving intoxicated individuals, prevention campaigns addressing young adults as the main users of E-scooters promoting proper personal protective equipment and highlighting the potential dangers of driving under the influence of alcohol, are imperative. As previously postulated, mandatory helmets should be taken into consideration by legislators.

Regarding the trauma surgeon’s perspective, a thorough examination, including generous imaging should be part of every consultation following E-scooter related injuries. Each assessment should include a neurological screening, backed by appropriate imaging, focusing on the head and neck area, as well as taking rare abdominal trauma into consideration.

## Limitations

The main limitation of this study is the unicentric design of a level 1 trauma center with a specialized department for Oral and Maxillofacial surgery, potentially leading to an overrepresentation of severely injured patients and patients suffering from facial trauma. Vice versa, minor injuries might be underreported. Additionally, not every patient was tested for blood alcohol, potentially distorting the results from our risk analysis.
